# Sirtuin 3 attenuates amyloid-β induced neuronal hypometabolism

**DOI:** 10.18632/aging.101592

**Published:** 2018-10-23

**Authors:** Junxiang Yin, Shiping Li, Megan Nielsen, Tanner Carcione, Winnie S. Liang, Jiong Shi

**Affiliations:** 1 Department of Neurology, Barrow Neurological Institute, St. Joseph Hospital and Medical Center, Dignity Health Organization, Phoenix, AZ 85013, USA; 2 Department of Neurology, the Second Hospital of Hebei Medical University, Shijiazhuang, China; 3 College of Science, University of Arizona, Tucson, AZ 85721, USA; 4 Translational Genomics Research Institute (TGen), Phoenix, AZ 85004, USA; 5 Advanced Innovation Center for Human Brain Protection, National Clinical Research Center for Neurological Diseases, Beijing Tiantan Hospital, Capital Medical University, Beijing, China; * Equal contribution

**Keywords:** Sirtuin 3, Alzheimer’s disease, cerebral hypometabolism, amyloid

## Abstract

Alzheimer’s disease (AD) is manifested by regional cerebral hypometabolism. Sirtuin 3 (Sirt3) is localized in mitochondria and regulates cellular metabolism, but the role of Sirt3 in AD-related hypometabolism remains elusive. We used expression profiling and weighted gene co-expression network analysis (WGCNA) to analyze cortical neurons from a transgenic mouse model of AD (*APPSwInd*). Based on WGCNA results, we measured NAD^+^ level, NAD^+^/ NADH ratio, Sirt3 protein level and its deacetylation activity, and ATP production across both in vivo and in vitro models. To investigate the effect of Sirt3 on amyloid-β (Aβ)-induced mitochondria damage, we knocked down and over-expressed Sirt3 in hippocampal cells. WGCNA revealed Sirt3 as a key player in Aβ-related hypometabolism. In APP mice, the NAD^+^ level, NAD^+^/ NADH ratio, Sirt3 protein level and activity, and ATP production were all reduced compared to the control. As a result, learning and memory performance were impaired in 9-month-old APP mice compared to wild type controls. Using hippocampal HT22 cells model, Sirt3 overexpression increased Sirt3 deacetylation activity, rescued mitochondria function, and salvaged ATP production, which were damaged by Aβ. Sirt3 plays an important role in regulating Aβ-induced cerebral hypometabolism. This study suggests a potential direction for AD therapy.

## Introduction

Alzheimer’s disease (AD) comprises about 60%–70% of all dementia cases [1]. Around 14–16 million Americans will be diagnosed with AD by 2050 unless new treatments are identified [2]. The incidence and burden of AD increase dramatically with age [3,4]. Though AD has been studied since 1906, the exact causes and pathogenic mechanisms remain to be elucidated.

Mounting evidence suggests that cerebral hypometabolism is strongly related with cognitive impairment in AD [5–7]. Not only is hypometabolism associated with impaired memory, but it is also thought as a predictor for the decline of a multitude of other cognitive functions. There is an inverse relationship between amyloid-β (Aβ) deposition and glucose metabolism [8]. Hypometabolism may be triggered by amyloid toxicity. Alternatively, hypometabolism can mediate and/ or exacerbate effects of amyloid toxicity. At preclinical stages, hypometabolism serves as an accurate predictor of cognitive decline and characterizes AD [9–11]. As a result, it is vital to investigate the mechanism of cerebral hypometabolism in AD. Multiple proteins within the mitochondria synergistically modulate Adenosine Triphosphate (ATP) production. Specifically, a key mitochondrial Nicotinamide adenine dinucleotide (NAD)^+^-dependent protein, Sirtuin 3 (Sirt3), plays an important role in energy metabolism [12,13]. In our previous studies, Sirt3 was reduced in postmortem AD brains and its reduction was associated with declined cognitive performance and increased severity of AD pathology. Sirt3 was downregulated by Aβ-42 in primary cultured neurons [14,15]. These studies suggest Sirt3 reduction plays an important role in pathogenesis of AD. However, little is known about the relationship between Sirt3-related energy regulation and hypometabolism in AD.

In this study, expression profiling and weighted gene co-expression network analysis (WGCNA) provided the evidence that Sirt3-related energy pathway plays an important role in neuronal hypometabolism. The role of Sirt3 in hypometabolism was subsequently investigated using *in vitro* and *in vivo* models. Our findings show that Sirt3 rescues Aβ-induced ATP reduction and mitochondrial damage.

## RESULTS

### Amyloid precursor protein (APP) regulated Sirt3 and its energy pathway.

**Figure 1:**
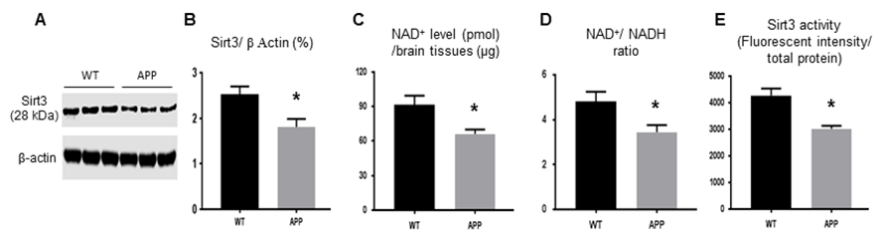
**The protein expression and activity of Sirt3 are reduced in APP mice.** Mouse fresh brain tissues were collected and homogenized. (**A, B**) Sirt3 protein expression by Western blot was lower in APP than WT mice. (**C**) NAD^+^ level and (**D**) NAD^+^/ NADH ratio were reduced in APP mice compared to WT mice. (**E**) Mitochondria were isolated from mouse brain to test Sirt3 deacetylation activity (ratio of fluorescent intensity to total protein). Sirt3 activity in APP mice was lower in APP mice than that in WT mice. Note: n=3 per group, * p<0.05.

WGCNA revealed that a list of molecules in energy metabolism changed significant in APP mice versus that in control mice including those that were closely related to the Sirt3 signal pathway (Table 1). In 9 month-old APP mouse brains, Sirt3 protein levels (1.81± 0.22) were significantly lower than that of the control (2.53± 0.17, p<0.05, Fig. 1A, B). The NAD^+^ level and NAD^+^/ NADH ratio were directly related with Sirt3 activity. NAD^+^ level (Fig 1C) and the NAD^+^/NADH ratio (Fig. 1D) showed statistically significant (p<0.05) decreases in APP mouse brains compared to controls. The deacetylation activity of Sirt3 in APP mice (3021± 116.3 unit/ per µg protein) was consistently reduced compared to that of the control mice (4273.7± 262.1 unit/ per µg protein, p< 0.01, Fig. 1E). In summary, Sirt3 protein expression, NAD^+^, NAD^+^/ NADH ratio, and Sirt3 activity were all down-regulated in APP mice.

**Table 1:** The effect of APP on gene expression of energy metabolism.

**Gene Name**	**log2 fold**	**p value**	**Description**	
PRKAA1	1.46493	0.0138586	protein kinase, AMP-activated, alpha 1 catalytic subunit	SIRT3 production
PPARG	-2.7667	0.0003537	peroxisome proliferator-activated receptor gamma	SIRT3 production
USP3	1.36399	0.0352496	ubiquitin specific peptidase 3	SIRT3 degradation
PSMD3	-1.9837	0.0037322	26S proteasome (prosome, macropain) non-ATPase regulatory subunit 3	SIRT3 degradation
ATPAF2	2.28485	0.0207859	ATP synthase mitochondrial F1 complex assembly factor 2	Energy metabolism
HMGCR	-0.9487	0.0230069	3-hydroxy-3-methylglutaryl-CoA reductase	Energy metabolism
ACSS2	1.47603	0.0004182	acyl-CoA synthetase short-chain family member 2	Energy metabolism
INSR	0.89617	0.0102131	insulin receptor	Energy metabolism
IRS1	1.54338	0.0389107	insulin receptor substrate 1	Energy metabolism
MTFR1L	1.7624	0.0326343	mitochondrial fission regulator 1-like	
TOMM20	1.29106	0.049985	translocase of outer mitochondrial membrane 20	

Note: PRKAA1, PPARG, USP3 and PSMD3 are involved in SIRT3 synthesis and metabolism.

### APP induced learning and memory deficits in mice

**Figure 2:**
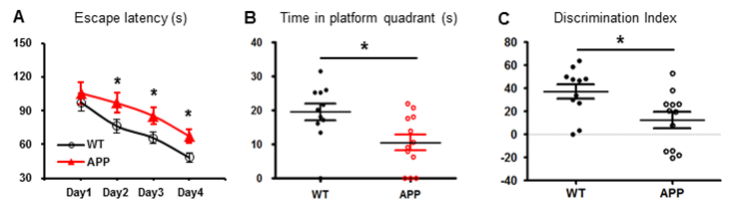
**Learning and memory is impaired in APP mice.** APP mice (n=11) and age-matched WT mice (n=12) were tested in MWM and NOR tests. (**A**) The escape latency was measured during the 4-day period. APP mice had a longer latency than WT mice. (**B**) The time spent in the target quadrant was measured on Day 5. APP mice spent much less time there than WT mice. (**C**) NOR discrimination index between APP and WT groups. *p< 0.05.

We next tested whether or not cerebral hypometabolism of APP mice is translated into poor performance in learning and memory. In Morris water maze (MWM) test, APP mice and age-matched WT mice had a similar baseline of escape latency on Day 1. During the four-day learning test, the escape latency of APP mice was increased on Day 2 and continued to increase as compared to WT mice (Fig. 2A). These data indicated that APP mice showed less learning ability. In the probe trial on Day 5, APP mice spent less time in the target quadrant compared to WT mice (Fig. 2B). APP mice showed memory deficit in comparison to WT mice. In the novel object recognition (NOR) test, APP mice spent less time with novel objects compared to WT mice (Fig. 2C). This result indicated that APP mice could not remember well enough to differentiate the old object from the new one.

### Sirt3 activity is downregulated by Aβ-42 *in vitro*

**Figure 3:**
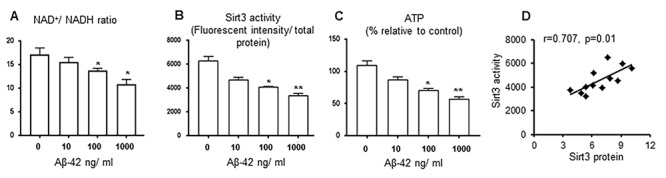
**Sirt3 activity is downregulated by Aβ-42 in vitro.** Primary cortical neurons were treated with Aβ-42 (0, 10,100,1000 ng/ ml) for 24 hours. (**A**) NAD^+^/ NADH ratio; (**B**) Mitochondrial Sirt3 deacetylation activity (the ratio of fluorescent intensity to total protein); (**C**) ATP levels were decreased when Aβ-42 concentration was increased. (**D**) The correlation between Sirt3 protein levels and its deacetylation activity. n=6 per group, * p< 0.05, **p< 0.01.

To test the effects of Aβ on Sirt3, we treated primary cortical neurons with oligomer Aβ-42 at varying concentrations. We chose oligomer Aβ-42 because it can freely enter neurons via a pore-forming mechanism, leading to subsequent calcium entry and mitochondrial damages [16–20]. Sirt3 protein levels were reduced in an Aβ-42 dose-dependent manner [14]. NAD^+^/ NADH ratio, as an important indicator of Sirt3 function, was decreased as the Aβ-42 concentration was increased (Fig. 3A). In isolated mitochondria, Sirt3 deacetylation activity was suppressed by Aβ-42 (Fig. 3B), so was the ATP production (Fig. 3C). The correlation analysis indicated that Sirt3 deacetylation activity was related with its protein levels in this study (Fig. 3D). These data provide evidence that Aβ-42 downregulated Sirt3 expression and impairs its function in primary neurons.

### Sirt3 rescues mitochondrial function impaired by Aβ-42

**Figure 4:**
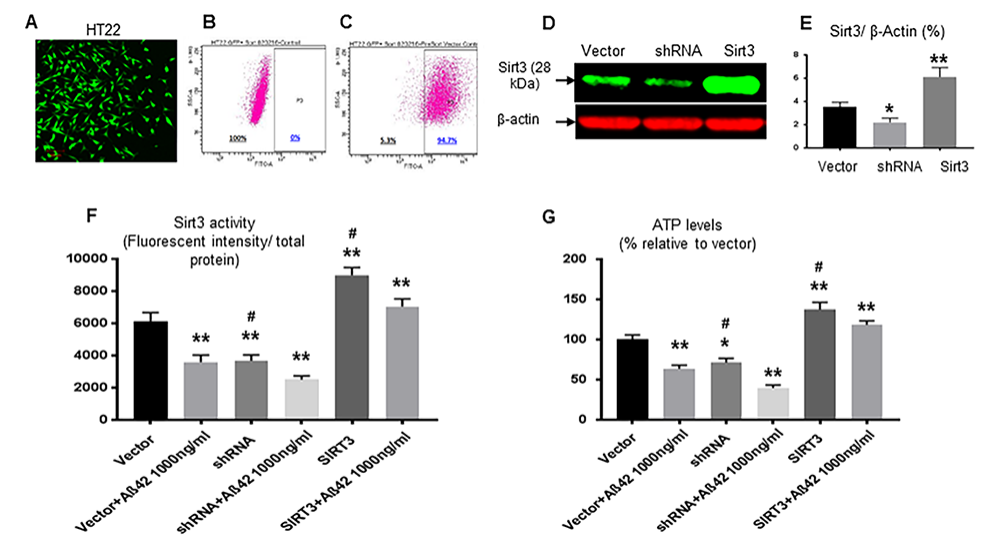
**Sirt3 rescues mitochondrial function impaired by Aβ-42.** (**A**) HT22 cells were successfully transfected (green) with vector, Sirt3 cDNA or shRNA. The percentage of transfection was analyzed on Accuri C6 Flow Cytometer. (**B**) Before transfection, the percentage was 0 (negative control); (**C**) After transfection, there were 94.7% cells transfected. Sirt3 protein levels in transfected cells were further confirmed by using Western blot. (**D**) A representative Western blot of Sirt3. β-actin was used as an internal control. (**E**) In transfected cells, Sirt3 protein levels were measured with normalization with β-actin. (**F**) Sirt3 deacetylation activity (the ratio of fluorescent intensity to total protein) and (**G**) ATP levels were tested in isolated mitochondria. N= 6 per group, *p< 0.05, **p< 0.01 vs. vector; #p< 0.05, ##p< 0.01 vs. respective treated group.

We further investigated whether Sirt3 overexpression could protect Aβ-42-induced mitochondria damages. Hippocampal HT22 cells were transfected by lentivirus containing Sirt3 cDNA (Sirt3 overexpression) or shRNA (Sirt3 knockdown) (Fig. 4A-C). Transfection was confirmed using Western blot [Sirt3 overexpression (6.1± 0.5%, p< 0.01), expression knock down (2.2± 0.2%, p< 0.05) vs. vector control (3.5±0.2%)] (Fig. 4D and E). Sirt3 deacetylation activity in isolated mitochondria was reduced in Sirt3 knockdown cells and increased in Sirt3 overexpressing cells. This activity was also reduced in Aβ-42 treated groups compared to their untreated counterpart. Compared to the vector control group (6148± 524), Sirt3 deacetylation activities were 3599± 428 (p< 0.01) in the vector treated with Aβ-42, 3682± 353 (p< 0.01) in shRNA cells, and 2506± 239 (p< 0.01) in shRNA treated with Aβ-42. Sirt3 deacetylation activity was increased in Sirt3 overexpression cells (8985± 475, p< 0.01) (Fig. 4F). This increased Sirt3 deacetylation activity was translated into enhancing mitochondrial function including the increased ATP production. Compared to the vector control group (100.2± 5.7), Aβ-42 treatment reduced intracellular ATP levels (63.5± 4.6, p< 0.01). Sirt3 knockdown showed further reduction [shRNA (71.3± 5.2, p< 0.05) and shRNA plus Aβ-42 (39.6±3.8, p< 0.01)]; whereas Sirt3 overexpression increased ATP levels by almost 40% (137.2± 9.2, p< 0.01, Fig. 4G).

## DISCUSSION

Cerebral hypometabolism is one of the early signs of AD and is thought to be a predictor of disease progression [5–7]. Although there is no evidence of chronic ischemia, regional cerebral glucose hypometabolism is consistent in AD patients and is correlated with the severity of dementia [21,22]. Since Aβ also occurs early in the course of the disease, there is a close relationship between Aβ deposition and reduced cerebral glucose metabolism [8]. Up to 40% of the effects of Aβ on episodic memory are attributable to hypometabolism as measured by FDG-PET [5]. Aβ treatment for 24 hours decreased ATP levels in human stem cell-derived neurons and astrocyte cultures. Interestingly, the NAD^+^/NADH ratio was increased first and then reduced [23]. The APP mouse model demonstrates amyloid deposition at 6 months and is ideal for investigating Aβ-induced hypometabolism.

Our previous study has demonstrated that Sirt3 is involved in AD pathology that is triggered by Aβ. This study further explored the relationship between Sirt3-related energy pathway and Aβ-induced hypometabolism in AD. Our data showed Sirt3 protein and its functions were significantly downregulated in both APP mice and Aβ-42-treated primary neurons. With genetic enhancement of its expression, Sirt3 overexpression partially rescued cellular energy metabolism damaged by Aβ-42.

WGCNA and pathway analysis of APP mice revealed several key molecules related to Sirt3. Peroxisome proliferator-activated receptor gamma coactivator-1α (PGC-1α) stimulates Sirt3 expression at both mRNA and protein levels [24], in an estrogen-related receptor α (ERRα)-dependent manner [25]. Co-transfection of PGC-1α and ERRα has a synergic effect on Sirt3 activity [24]. The mRNA of peroxisome proliferator-activated receptor gamma in APP mouse brain was reduced compared to WT mice brains (Table 1), suggesting the production of Sirt3 may be down-regulated in APP mice. The ubiquitin proteasome pathway (UPP) is responsible for degradation of intracellular proteins. The 26S proteasome is distributed throughout eukaryotic cells at a high concentration and cleaves peptides in an ATP/ ubiquitin-dependent process. Evidence indicates that Sirt3 is ubiquitinated and the E3 ubiquitin ligase is involved in Sirt3 degradation [26,27]. Loss of ubiquitin-specific peptidase 3 **(**USP3) increased the levels of histone ubiquitination in adult tissues, and shortened animal life span [28]. In APP mouse brains, USP3 expression was significantly higher than WT mice, suggesting that the degradation of Sirt3 may be up-regulated in APP mice. Sirt3 deacetylates several mitochondrial proteins, including long-chain acyl-CoA dehydrogenase, Acetyl-coenzyme A synthetase 2 (AceCS2) and 3-hydroxy-3-methylglutaryl CoA synthase 2. Deacetylation of AceCS2 by Sirt3 activates the acetyl-CoA synthetase activity of AceCS2 [29]. Thus, Sirt3 deacetylation activity can affect the ATP energy pathway. Mitochondrial ATP synthase is a large protein complex located in the mitochondrial inner membrane, where it catalyzes ATP synthesis. ATP synthase mitochondrial F1 complex assembly factor 2 (ATPAF2) facilitates the biogenesis of ATP synthase [30]. In APP mouse brain, ATPAF2 expression was down-regulated in comparison to WT mice. This change suggests that the Sirt3-related energy pathway may play an important role in ATP production.

Pursuing the lead following WGCNA and pathway analysis, we systemically studied the effects of Aβ on changes in Sirt3 expression and activity both in vivo and in vitro. We found that NAD^+^, NAD^+^/NADH ratio, Sirt3 protein levels and activities were noticeably reduced in APP mouse brain compared to WT. By genetic modification, Sirt3 knockdown showed further decline in ATP production, whereas Sirt3 overexpression helped to rescue Aβ-induced hypometabolism.

The association between Aβ toxicity, mitochondrial dysfunction, oxidative stress and neuronal death has been well studied. Aβ increases production of mitochondrial ROS [31] and mitochondrial Ca^++^ mobilization may contribute to this process [32]. Because mitochondrial DNA is inherited maternally, brain hypometabolism is more pronounced in AD patients who have a maternal family history of AD than those with a paternal family history or those with a negative family history [33].

Sirt3 is localized in mitochondria and plays a critical role in ATP production. The Sirt3 signal pathway has been studied in neurodegenerative diseases. β-hydroxybutyrate, a potent Sirt3 agonist, blocked the entry of Aβ into the neurons and restored mitochondria functions, which attenuated cognitive decline in this APP mouse model of AD [16]. In animal models of hypertension, cardiac disease and ischemic stroke, Sirt3 has shown to suppress oxidative stress by regulating NOX2, NOX4, SOD2, Nrf2 and FoxO3a [34–37].

In conclusion, these data suggest that Sirt3 plays an important role in pathophysiology at an early stage of development of AD. The rescuing effects of Sirt3 on mitochondria and ATP production may provide a potential disease-modifying target for AD therapy.

## MATERIALS AND METHODS

### Animals

All experiments involving animals were performed following protocols in accordance to the Revised Guide for the Care and Use of Laboratory Animals that was approved by the Institutional Animal Care and Use Committee (IACUC) of the Barrow Neurological Institute.

J20 transgenic mice were purchased from the Jackson Laboratory (Bar Harbor, ME). These transgenic mice express a mutant form of the human APP. They have both the Swedish (K670N/M671L) and the Indiana (V717F) mutations (*APPSwInd*). Diffuse Aβ deposition starts to appear in the dentate gyrus and neocortex at age 5-7 months. It is progressive with all transgenic mice exhibiting plaques by age 8-10 months [38]. We used 9 month-old APP mice and age-matched wild type (WT) mice with the same B6 background (12-14 animals per group).

### Behavioral studies

The MWM was used to test spatial learning and memory function as described previously [39]. Mice were given 6 trials per day for 4 days, with an inter-trial interval of 20 minutes. A single probe trial was carried out 24 hours after the hidden platform task had been completed. Medial temporal lobe and frontal lobe function was assessed using the NOR test [40]. Twenty-four hours prior to the NOR test, mice were habituated to the empty open field for 10 minutes. During training, mice were placed in the center of the open field containing two identical objects, and the time spent exploring each object during a 5-minute period was quantified. Four hours later, the mice were tested by replacing one of the identical objects with a novel object of different shape and color. Novel object recognition was quantified using the discrimination index (DI) calculated as: DI= (Exploration Time for Novel object - Exploration Time for Familiar object)/ Total Exploration time.

### Preparation of mouse brain tissue

Mice were euthanized by injection of a ketamine/xylamine cocktail at 9 months. Fresh brain tissues (temporal cortex) were quickly collected on ice. Parts of fresh brain tissues were used immediately for NAD^+^ and NADH measurement, Sirt3 deacetylation activity test after mitochondrial isolation. The fresh temporal cortex was stored at −80°C for Sirt3 protein level test in western blotting. For weighted gene co-expression network analysis, mice brains were harvested and frozen, sectioned (8 μm), and fixed on glass slides.

### Weighted Gene Co-expression Network analysis

Mouse brain samples (n=6 in each group) were used for transcriptomic analyses as described previously [41]. Brain sections were stained with a combination of thioflavin-S (Sigma-Aldrich, Dallas, TX) and 1% neutral red (Fisher Scientific, Chicago, IL). Pyramidal neurons were detected and laser-captured. Total RNA was isolated using the Arcturus PicoPure RNA Isolation Kit with DNase I treatment (Qiagen, Valencia, CA). Isolated total RNA from each sample of approximately 500 neurons was double-round amplified, cleaned, and biotin-labeled with Affymetrix’s GeneChip per the manufacturer’s protocol. Amplified and labeled complementary RNA was quantified on a spectrophotometer and run on a 1% Tris-acetate-EDTA gel to check for an evenly distributed range of transcript sizes.

*Data analysis*: Following completion of sequencing, we initiated an automated in-house pipeline for NGS analysis through our laboratory database. BCL conversion was performed using Illumina’s BCL Converter and FASTQ alignments was aligned using STAR [42]. Alignment produced single BAM files containing both aligned and unaligned data. DEseq2 was used to identify differentially expressed transcripts and p-values was corrected using the Benjamini and Hochberg False Discovery Rate [43]. Gene annotation was performed based on Gencode version 3 (ENSEMBL) and build 37.1. WGCNA was performed on differentially expressed genes (corrected p < 0.05) [44,45]. Gene sets were loaded into the WGCNA R package, and annotation of generated modules was performed using DAVID [46]. We chose genes that are known to be Sirt3 related in the energy metabolism pathway.

### Primary neuron cultures

Primary cortical neuron cultures were prepared from new-born mouse pups as described previously [14,34]. Briefly, cortical neurons grew on poly-D-lysine coated dishes in neuronal culture media. On day 14^th^, cultured neurons were treated with Aβ-42 (0, 10,100,1000ng/ml) for 24 hours, and then further measures were carried out as described in the text.

### Preparation of oligomeric Aβ-42

Human synthetic Aβ-42 (rPeptide) was treated in 20 µM ammonium acetate in distilled water and incubate for 30 minutes at room temperature, then lyophilized and stored at -80^0^C. The pretreated Aβ-42 was dissolved in artificial cerebrospinal fluid (aCSF) or culture media at room temperature immediately prior to use. The presence and stability of oligomers was tested by electron microscopy and western blot techniques [14, 16).

### Sirt3 vector construction and transfection

Sirt3 was either over-expressed or knocked down as described previously [14,34] with modifications in hippocampal HT22 cells. HT22 cells grew in DMEM medium (ThermoFisher Scientific, #12491015) plus 10% fetal bovine serum and 1% penicillin-streptomycin-glutamine (ThermoFisher Scientific, #10378-016) in humidified incubator (37C, 5%CO2). After cells grew 70% confluent in 6 wells plate, fresh media with lentiviral particles (multiplicity of infection =20) was added. On next day, media with lentiviral particles was removed and replaced fresh media. Then, media with puromycin (3ug/ml) was added and replaced media with puromycin every 3-4 days. HT22 cells with successfully transfected with vector, Sirt3 or shRNA containing green fluorescent protein (GFP) were checked under fluorescent microscope. Transfected HT22 cells grew in media with puromycin (3ug/ml) and were treated with Aβ-42 for 24 hours; and then further measures were carried out as described in the text.

### Mitochondrial isolation

Mitochondria were isolated from cells or fresh brain tissues as we described previously [15,34]. Cells/ tissues were collected and was homogenized using dounce homogenizer (for cells) or glass homogenizer (for tissue) in mitochondrial isolation buffer (320 mM sucrose, 1 mM EDTA, and 10 mM Tris-HCl, pH=7.4) on ice. The homogenate was centrifuged 1000×g at 4°C for 10 min and the supernatant was collected. The supernatant was spin for 20 min at 13000×g at 4°C. The resulting pellet was resuspended in mitochondrial respiration buffer (110 mM sucrose, 0.5 mM EGTA, 3mM MgCl2, 40 mM KCl, 10 mM KH2PO4, 20 mM HEPES, 1g/l BSA) to make mitochondrial sample solution.

### NAD^+^ and NADH measurement

NAD^+^, NADH measurement in tissues or cells was carried out as described previously [14,34]. Total NAD^+^ and NADH were tested using an NAD^+^/ NADH assay kit (ab65348, Abcam, Cambridge, MA) according to the manufacturer’s protocol. NAD^+^/ NADH ratio was calculated based on the value of total NAD^+^ and NADH (NAD^+^= total NAD^+^– NADH).

### ATP level measurement in cultured cells

ATP levels were measured as described previously [14]. Cultured cells were seeded in a 96-well plate (2.5 x104 cells per well, n= 6 wells per group) and treated with oligo Aβ-42 for 24 hours before further measurement. ATP levels were measured using a Luminescent ATP detection assay kit (#ab113849, Abcam, Cambridge, MA) according to the manufacturer’s protocol. The ATP value of vector cells without Aβ-42 treatment was normalized to 100%.

### Mitochondrial Sirt3 deacetylation activity

After mitochondria were isolated from cells or brain tissues, Sirt3 deacetylation activity was measured using Sirt3 activity assay kit (#ab156067, Abcam, Cambridge, MA) following the manufacturer’s protocol. The fluorescent intensity was collected on Tecan infinity M200 pro according to the manufacturer’s protocol and was normalized with the amount of total test protein. Final data were normalized with total protein concentration from cells or tissues.

Sirt3 deacetylation activity was represented as the ratio of fluorescent intensity to the amount of total test protein and the ratio was used for statistical analysis.

### Western blot

Fifty µg protein was used for Western blotting. Primary antibodies were the following: anti-Sirt3 (#5490S, Cell Signaling, Danvers, MA), anti-β-actin (Santa Cruz, Dallas, TX), IRDye 800CW and IRDye 680CW antibodies (LI-COR, Lincoln, NE). Immunoreactivity signals were quantified using Odyssey CLx (LI-COR, Lincoln, NE). Protein levels were presented as a percentage relative to β-actin, an internal control.

### Statistical analysis

Data are expressed as mean± SEM. An unpaired Student’s T-test was used for comparison between the two groups to determine the significance of the difference by using the GraphPad Prism 4.0 software. A value of p< 0.05 was considered significant.
